# Synthesis and Antioxidant Activities of Novel 5-Chlorocurcumin, Complemented by Semiempirical Calculations

**DOI:** 10.1155/2013/354982

**Published:** 2013-09-21

**Authors:** Ahmed A. Al-Amiery, Abdul Amir H. Kadhum, Hasan R. Obayes, Abu Bakar Mohamad

**Affiliations:** ^1^Department of Chemical & Process Engineering, Universiti of Kebangsaan Malaysia (UKM), 43000 Bangi, Selangor, Malaysia; ^2^Applied Chemistry Division, Applied Science Department, University of Technology (UOT), Baghdad 10001, Iraq

## Abstract

The novel curcumin derivative (1E,4Z,6E)-5-chloro-1,7-bis(4-hydroxy-3-methoxyphenyl)hepta-1,4,6-trien-3-one (5-chlorocurcumin) was prepared from natural curcumin. The newly synthesised compound was characterised by spectral studies (IR, 1H NMR, and 13C NMR). The free radical scavenging activity of 5-chlorocurcumin has been determined by measuring interaction with the stable free radical DPPH, and 5-chlorocurcumin has shown encouraging antioxidant activities. Theory calculations of the synthesised 5-chlorocurcumin were performed using molecular structures with optimised geometries. Molecular orbital calculations provided a detailed description of the orbitals, including spatial characteristics, nodal patterns, and the contributions of individual atoms.

## 1. Introduction

**Figure 1:**
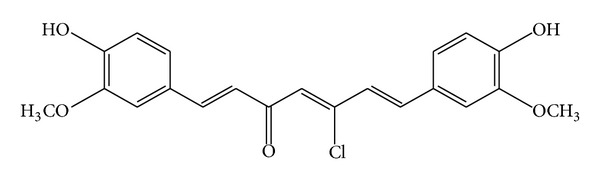
Structure of 5-chlorocurcumin.

 Curcumin (1) [diferuloylmethane, 1,7-bis(4-hydroxy-3-methoxyphenyl)-1,6-hepta-diene-3,5-dione] is a well-known acyclic diarylheptanoid identified as the major constituent of turmeric powder extracted from the rhizome of the plant *Curcuma longa* [1, 2]. Curcumin (1) has potent antioxidant activity [3–5] and has received attention as a promising nutraceutical or as a component of designer foods for its cancer preventive ability. With a unique conjugated structure including two methoxylated phenols and an enol *β*-diketone, curcumin shows a typical radical-trapping ability as a chain-breaking antioxidant. The antioxidant mechanism of curcumin and curcumin-related phenols has attracted significant attention [6–8], but it is still not well understood. In addition, curcumin blocks growth factor signaling via inhibition of tyrosine kinase activity or depletion of ErbB-2 [9]. More recently, it has been shown that curcumin causes cleavage of h-catenin, resulting in apoptosis in a colon cancer—derived cell line [10]. Antioxidant compounds play an important role as a health protecting factor. Scientific evidence suggests that antioxidants reduce the risk for chronic diseases including cancer and heart disease. Primary sources of naturally occurring antioxidants are whole grains, fruits, and vegetables. Plant sourced food antioxidants like vitamin C, vitamin E, carotenes, phenolic acids, phytate, and phytoestrogens have been recognized as having the potential to reduce disease risk. Most of the antioxidant compounds in a typical diet are derived from plant sources and belong to various classes of compounds with a wide variety of physical and chemical properties. Some compounds, such as gallates, have strong antioxidant activity, while others, such as the monophenols, are weak antioxidants [11]. For instance, in the DPPH (2,2-diphenyl-1-picrylhydrazyl) analysis the antioxidant properties of compounds change as follows: gallic acid > caffeic acid ~ ascorbic acid ~ Trolox > sinapinic acid > isoeugenol [12]. ABTS (2,20-azinobis(3-ethylbenzothiazoline-6-sulfonic acid) assay has shown that the sinapinic acid is a weaker antioxidant than the gallic acid but stronger than the rest of the tested compounds [12]. crocin bleaching assay (CBA) test has shown that the sinapinic acid is the strongest antioxidant of all investigated compounds [12]. In Oxygen Radical Absorbance Capacity (ORAC) assay only caffeic acid and isoeugenol have stronger antioxidant abilities than the sinapinic acid [12]. Free radicals are very reactive chemical species with an unpaired electron. Because of their reactivity lipids, proteins, and DNA can be damaged by the radicals action. In consequence, they are responsible for many diseases such as cancer [13–16], cardiovascular disorders [17–21], atherosclerosis [22–24], and asthma, arthritis, neurodegenerative disorders: Alzheimer's [25–29], Parkinson's diseases, and dementia [30]. In the present study, the new curcumin derivative (1E,4Z,6E)-5-chloro-1,7-bis(4-hydroxy-3-methoxyphenyl)hepta-1,4,6-trien-3-one (5-chlorocurcumin) was synthesised, and a structure was proposed based on spectroscopic evidence (Figure 1) and in view of the considerable importance of the curcumin as antioxidant the present work is also aimed for testing of 5-chlorocurcumin for free radical scavenging activity by using DPPH free radical scavenging methods.

## 2. Experimental Section

### 2.1. Chemistry

All chemicals used were of reagent grade (supplied by Malaysia *|* Sigma-Aldrich) and were used as received without further purification. The Fourier transform infrared (FTIR) spectra were measured using a Thermo Scientific Model Nicolet 6700 spectrophotometer. NMR spectra were recorded on a Model AVANCE III 600 MHz spectrometer. 

#### 2.1.1. Synthesis of 5-Chlorocurcumin

Curcumin (1.23 g, 0.00334 Mol) was mixed with POCl_3_ (10 mL), and the resulting suspension was refluxed for approximately 3 hours. The reaction was then cooled to room temperature and slowly poured onto crushed ice mixed with water. The violet solid formed was collected by filtration, washed with ice water, and recrystallised from acetonitrile. 

#### 2.1.2. Theoretical Studies

The molecular representation of the reference compound was plotted using ChemBioOffice 2010 software. All the quantum chemical calculations were performed using the Austin Model 1 (AM1), Parameterized Model number 3 (PM3), and Modified Neglect of Differential Overlap (MNDO) methodology, while the molecular atomic charges were calculated via the Mulliken charges [31].

### 2.2. Evaluation of Antioxidant Activity

Stock solution (1 mg/mL) was diluted to final concentrations of 20–100 *μ*g/mL. Ethanolic DPPH solution (1 mL, 0.3 mmol) was added to sample solutions in DMSO (3 mL) at different concentrations (50–300 *μ*g/mL) [32]. The mixture was shaken vigorously and allowed to stand at room temperature for 30 min. The absorbance was then measured at 517 nm in a UV-Vis spectrophotometer. The lower absorbance of the reaction mixture indicates higher free radical scavenging activity. Ethanol was used as the solvent and ascorbic acid as the standard. The DPPH radical scavenger was calculated using the following equation:

(1) $$\begin{array}{c} \text{Scavenging}\;\text{effect}=\frac{A_{0}-A_{1}}{A_{0}}\times 100, \end{array}$$

where *A*_0_ is the absorbance of the control reaction and *A*_1_ is the absorbance in the presence of the samples or standards *A*_0_ − *A*_1_.

## 3. Results and Discussion

### 3.1. Chemistry

The synthesis of (1E,4Z,6E)-5-chloro-1,7-bis(4-hydroxy-3-methoxyphenyl)hepta-1,4,6-trien-3-one (5-chlorocurcumin) was conducted via the reaction of curcumin with phosphorus oxychloride. For the FTIR spectrum of 5-chlorocurcumin, the broad peak at 3412.9 cm^−1^ was due to O–H stretching vibration, while the sharp peak at 1727.8 was due to carbonyl stretching. Peaks at 2959.7 cm^−1^ and 2931.6 cm^−1^ were assigned to aromatic rings on 5-chlorocurcumin. Peaks at 1598.8 cm^−1^ and 1513.0 cm^−1^ were typical of C=C stretch vibrations. ^1^H NMR (CDCl_3_): *δ* (ppm) 7.5 (H–C=C); 7.27 (H-aromatic); 4.2 (–OH); 0.9 (–OCH_3_). ^13^C NMR (CDCl_3_): *δ* (ppm) 167.7 (C=O); 128–132 (C-aromatic); 68.18 (C–Cl); 38.8 (–OCH_3_).

### 3.2. Computational Studies

#### 3.2.1. Mulliken Charges

**Figure 2:**
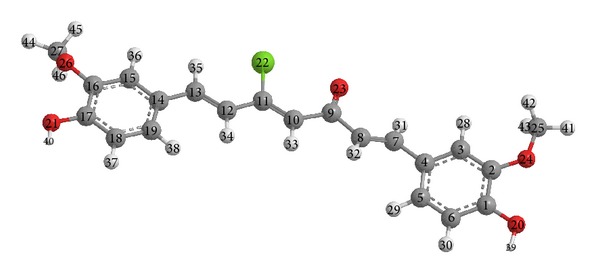
Optimised 3D geometrical structure for 5-chlorocurcumin.

An earlier study [33–37] had shown that atomic charges were affected by the substituents present on rings. With the aid of a reference model, a 5-chlorocurcumin structure with optimised geometries and 3D geometrical structures is shown in Figure 2. The data showed that the highest atomic charge (Table 1) by AM1, PM3, and MNDO was [O_23_  −0.2950], [O_23_  −0.3273], and [O_23_  −0.2891], respectively, followed by [C_8_  −0.2405], [C_8_  −0.2367], and [O_24_  −0.2779]. These data clearly showed that these atoms were most reactive toward substitution reactions. The determined bond and twist angles, stretch, bend, stretch-bend, torsion, and the 3D geometrical structure indicated that this molecule was a nonplanar molecule with the stereochemistry C(7)-C(8): (E); C(10)-C(11): (Z); and C(12)-C(13): (E).

#### 3.2.2. AM1, PM3, and MNDO

**Figure 3:**
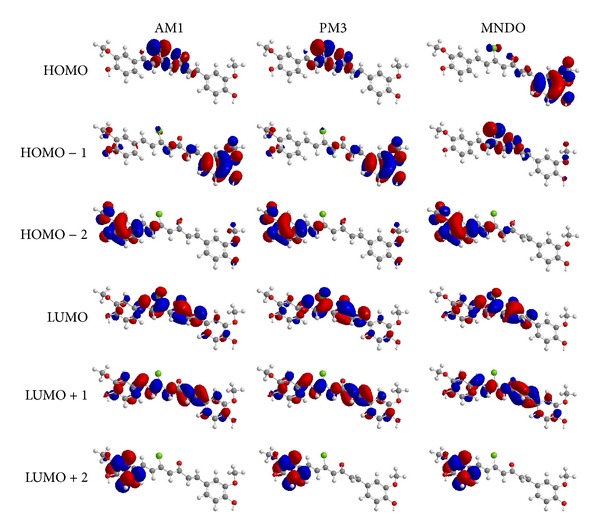
The highest occupied molecular orbital (HOMO) and the lowest unoccupied molecular orbital (LUMO) of 5-chlorocurcumin.

AM1, PM3, and MNDO calculations were performed for 5-chlorocurcumin. The optimised molecular structure of the most stable form is shown in Figure 1, and the calculated energies are presented in Table 2. Molecular orbital calculations provided a detailed description of the orbitals including the spatial characteristics, nodal patterns, and individual atom contributions. The contour plots of the frontier orbitals for the ground state of 5-chlorocurcumin are shown in Figure 3 together with the highest occupied molecular orbital (HOMO) and the lowest unoccupied molecular orbital (LUMO). It was interesting that both orbitals were substantially distributed over the conjugation plane.

In addition, it can be observed in Figure 3 that the HOMO orbitals were located on the substituted molecule, while the LUMO orbitals resembled those obtained for the unsubstituted molecule. Therefore, the substitution influenced the electron donation ability while imposing only a small impact on the electron acceptance ability. 

The orbital energy levels of the HOMO and LUMO of 5-chlorocurcumin are listed in Table 3. An electronic system with a larger HOMO-LUMO gap should be less reactive than one having a smaller gap. In the present study, the HOMO-LUMO gap values of 5-chlorocurcumin by AM1, PM3, and MNDO methods were −5.109, −4.642, and −5.944 eV, respectively. The lower value in the HOMO and LUMO energy gap would explain the eventual charge-transfer interaction taking place within the molecules. The low HOMO values for 5-chlorocurcumin indicated that this molecule had low ionisation energies, suggesting that it could lose electrons easily. These results indicated that 5-chlorocurcumin was potentially a good antioxidant.

The UV-Vis absorption spectrum of 5-chlorocurcumin was recorded in ethanol. Absorption peaks were observed at 255, 281, and 419 nm for 5-chlorocurcumin. These peaks were assumed to be the n→p* and p→p* transitions. The 3D plots of the HOMO−2, HOMO−1, HOMO, LUMO, LUMO+1, and LUMO+2 and the corresponding energy levels for 5-chlorocurcumin are shown in Figure 3. The theoretical electronic transfers (ET) for 5-chlorocurcumin by the AM1, PM3, and MNDO methods were at 242.677, 267.092, and 208.587 nm, corresponding to the UV-Vis spectral absorption peaks and the electronic transfers of HOMO plus LUMO. 

Figure 3 shows the six main orbitals contributing to the vertical electronic transitions of 5-chlorocurcumin. These orbitals, HOMO−2, HOMO−1, HOMO, LUMO, LUMO+1 and LUMO+2, represent the three highest occupied orbitals and the three lowest unoccupied orbitals in 5-chlorocurcumin. Similar spatial distributions of orbitals between HOMO/HOMO−1/HOMO−2 and LUMO/LUMO+1/LUMO+2 pairs and the population analysis for 5-chlorocurcumin indicated that the electronic transitions and the electron clouds of the HOMO were delocalised on the carbonyl, chloride and also on carbon-carbon double bond (C10-C11) for AM1 and PM3 but delocalized on the benzene ring for MNDO. However, HOMO−1 was delocalised on the benzene ring for AM1 and PM3 but delocalised on the carbonyl, chloride and also on carbon-carbon double bond (C10-C11) for MNDO. Meanwhile, the HOMO−2 was delocalised on the benzene ring for all methods and also on carbon-carbon double bond (C10-C11) for AM1 and PM3. These orbitals were p-type bonding orbitals.

For comparison, the LUMO was found to be mainly delocalised on the carbonyl and chloride groups, while LUMO+1 was delocalised on the carbonyl, chloride and benzene ring. The LUMO+2 was mainly delocalised on the benzene ring. These orbitals were p-type bonding orbitals. Finally, the LUMO was found to be mainly delocalised on the benzene ring. In all cases, the LUMOs exhibited p*-type antibonding orbital characteristics.

### 3.3. Radical Scavenging Activity

DPPH is a relatively stable nitrogen-centered free radical that easily accepts an electron or hydrogen radical to become a stable diamagnetic molecule. DPPH radicals react with suitable reducing agents as a result of which the electrons become paired off forming the corresponding hydrazine. The solution therefore loses colour stoichiometrically depending on the number of electrons taken up. Substances capable of donating electrons/hydrogen atoms are able to convert DPPH (purple) into their nonradical form 1, 1-diphenyl-2-picrylhydrazine (yellow), a reaction which can be followed spectrophotometrically. From results, it may be postulated that 5-chlorocurcumin was able to reduce the stable free radical DPPH to the yellow-coloured diphenylpicrylhydrazine exhibiting better free radical scavenging activity than the curcumin itself and standard antioxidant ascorbic acid. Structure activity relationship study showed that the antioxidant activity of 5-chlorocurcumin could be attributed to electron donating nature of the substituents like –OH and –Cl on 5-chlorocurcumin scaffold, reduce free radical DPPH, and prevent the damage of cell. The more the hydrogen donors are, the stronger the antioxidant activity is. These antioxidants should display antioxidant activity, if one or more groups like –OH and –CH_3_ are free, since they are known to be good hydrogen donors [38, 39]. The DPPH radical assay provides an easy and rapid way to evaluate the antiradical activities of antioxidants. Determination of the reaction kinetic types DPPHH is a product of the reaction between DPPH^∙^ and an antioxidant:

(2) $$\begin{array}{c} \left(\text{AH}\right)\text{: }{\text{DPPH}}^{∙}+\;\text{AH}\to \text{DPPHH}+{\text{A}}^{∙} \end{array}$$

**Figure 4:**
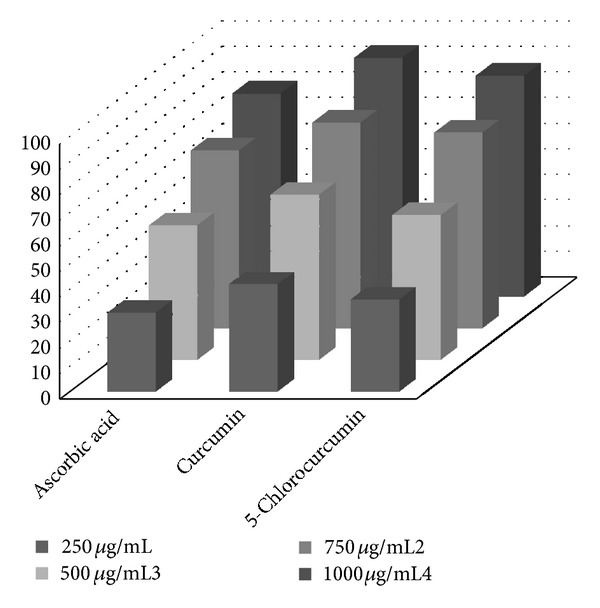
Scavenging effect of 5-chlorocurcumin, curcumin, and ascorbic acid at different concentrations (15, 30, 45, 60, 80, and 100 *μ*g/mL).

The reversibility of the reaction is evaluated by adding DPPHH at the end of the reaction. If there is an increase in the percentage of remaining DPPH^∙^ at the plateau, the reaction is reversible; otherwise it is a complete reaction. DPPH was used as a stable free radical electron that accepts an electron or hydrogen radical to become a stable diamagnetic molecule [40]. DPPH is a stable free radical containing an odd electron in its structure and usually used for detection of the radical scavenging activity in chemical analysis [41]. The reduction capability of DPPH radicals was determined by decrease in its absorbance at 517 nm induced by antioxidants [42]. Graph was plotted with percentage scavenging effects on the *y*-axis and concentration (*μ*g/mL) on the *x*-axis. The scavenging ability of the 5-chlorocurcumin was compared with ascorbic acid as a standard. 5-Chlorocurcumin showed good activities as a radical scavenger compared with curcumin and ascorbic acid (Figure 4).

There are postulated mechanisms for the reaction of 5-chlorocurcumin as an antioxidant which depends on the hydrogen atoms (hydroxyl groups O20 and O21), where these atoms were under the influence of two effects, namely, resonance and inductive. The resonance effect of 5-chlorocurcumin makes the release of hydrogen a free radical easy, while the inductive effect on benzene ring and methoxy group pushes the electrons toward oxygen free radical, resulting in the molecule becoming stable. The postulated mechanism depends on five known antioxidant mechanisms which describe antioxidant reactions [43–45].

**Scheme 1:**
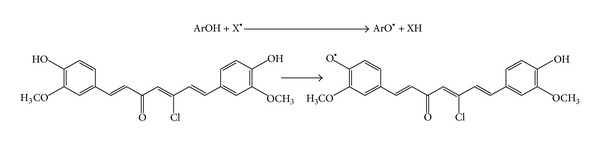
Hydrogen atom transfer mechanism.

*First Mechanism Is Hydrogen Atom Transfer *(*HAT*). Determining how antioxidants inherently donate their hydrogen atom to radicals, the environment in which they do this is also an important mechanistic consideration. For example, a solvent can affect the rate of hydrogen atom donation for a phenol by hydrogen bonding to the phenolic hydrogen, and in general, those solvents that have strong hydrogen bond accepting groups, that is, carbonyls or nitriles, tend to slow the rate of hydrogen atom donation substantially. This phenomenon supports a hydrogen atom-transfer mechanism since electron transfer would be facilitated under these conditions. Phenolic antioxidant reacts directly with a free radical which is neutralized, and a radical form of phenolic antioxidant appears. A numerical parameter associated with this mechanism is bond-dissociation enthalpy (BDE). The lower BDE parameter characterizes better antioxidant property. According to the Mulliken charges for 5-chlorocurcumin the hydroxyl group near the carbonyl (O_20_) can release *H* better than the one near chloro atom (O_21_) (Scheme 1).

**Scheme 2:**
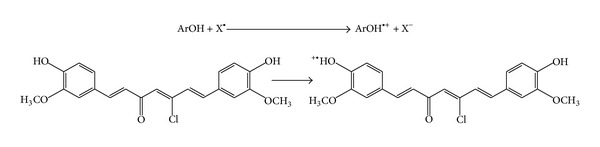
Single electron transfer mechanism.

*Second Mechanism Is Single Electron Transfer (SET). *SET mechanism measures the ability of a potential antioxidant to transfer one electron to reduce compounds such as radicals, metals, and carbonyls by a reduction of a coloured oxidant as in the ABTS, DPPH, and FRAP assays [46]. SET assays measure the capacity of an antioxidant to reduce an oxidant which changes colour when reduced. The degree of colour change is correlated with the antioxidant activity. SET reactions are pH-dependent and relatively slow and can require a long time to reach completion. Antioxidant capacity is based on the relative percent of the decrease in product rather than kinetics [47]. A numerical parameter related to the SET mechanism is adiabatic ionization potential (AIP), (Scheme 2). 

## 4. Conclusions

In this study, 5-chlorocurcumin was synthesized by the reaction of curcumin with phosphorus oxychloride. 5-Chlorocurcumin was characterized using various spectroscopic methods (FT-IR and NMR). The synthesised compound was studied theoretically, and the atomic charges and stereochemistry were estimated. The title compound was revealed to be nonplanar. The synthesized 5-chlorocurcumin was tested for antioxidant activity and found to be a superior antioxidant compound as compared to curcumin and ascorbic acid. 

## Figures and Tables

**Table 1:** Mulliken charges for 5-chlorocurcumin using AM1, PM3, and MNDO methods.

Atoms	AM1/charges	PM3/charges	MNDO/charges
C(1)	0.0738	0.0838	0.1059
C(2)	0.0537	0.0527	0.1091
C(3)	−0.1560	−0.1338	−0.0771
C(4)	−0.0681	−0.0772	−0.0744
C(5)	−0.0968	−0.0714	−0.0203
C(6)	−0.1977	−0.1786	−0.1330
C(7)	−0.0352	0.0016	0.0425
C(8)	−0.2405	−0.2367	−0.1555
C(9)	0.2857	0.3486	0.2915
C(10)	−0.2361	−0.2355	−0.1313
C(11)	0.0239	−0.0453	0.0916
C(12)	−0.1694	−0.1570	−0.0985
C(13)	−0.0631	−0.0429	0.0287
C(14)	−0.0687	−0.0720	−0.0827
C(15)	−0.1365	−0.1180	−0.0437
C(16)	0.0406	0.0394	0.0798
C(17)	0.0803	0.0896	0.1196
C(18)	−0.2025	−0.1787	−0.1413
C(19)	−0.0895	−0.0718	−0.0027
O(20)	−0.2250	−0.2039	−0.2256
O(21)	−0.2243	−0.2029	−0.2266
Cl(22)	0.0111	0.1126	−0.1004
O(23)	−0.2950	−0.3273	−0.2891
O(24)	−0.1940	−0.1723	−0.2779
C(25)	−0.0772	0.0473	0.2150
O(26)	−0.1870	−0.1655	−0.2723
C(27)	−0.0803	0.0457	0.2157
H(28)	0.1463	0.1225	0.0673
H(29)	0.1369	0.1087	0.0601
H(30)	0.1378	0.1113	0.0643
H(31)	0.1480	0.1160	0.0646
H(32)	0.1330	0.1151	0.0603
H(33)	0.1399	0.1176	0.0654
H(34)	0.1355	0.1171	0.0582
H(35)	0.1389	0.1119	0.0605
H(36)	0.1450	0.1211	0.0684
H(37)	0.1385	0.1120	0.0659
H(38)	0.1362	0.1105	0.0594
H(39)	0.2159	0.1946	0.1915
H(40)	0.2167	0.1947	0.1940
H(41)	0.1083	0.0532	0.0156
H(42)	0.0750	0.0279	−0.0136
H(43)	0.0653	0.0247	−0.0189
H(44)	0.1100	0.0534	0.0195
H(45)	0.0732	0.0285	−0.0150
H(46)	0.0732	0.0285	−0.0146

**Table 2:** Total energy and heat of formation of 5-chlorocurcumin.

Method	Total energy (Kcal/Mol)	Heat of formation (Kcal/Mol)
AM1	−113534.8813	−99.534
PM3	−105232.4967	−104.2199
MNDO	−113377.2376	−110.2337

**Table 3:** HOMO and LUMO energies (eV) of 5-chlorocurcumin.

Method	HOMO	LUMO	Δ*E*	HOMO − 1	LUMO + 1	Δ*E*	HOMO − 2	LUMO + 2	Δ*E*
AM1	−10.845	−5.736	−5.109	−11.189	−2.871	−8.318	−11.531	−0.291	−11.24
PM3	−10.461	−11.082	−4.642	−11.477	−5.819	−7.815	−3.267	−0.252	−11.225
MNDO	−10.962	−11.086	−5.944	−11.364	−5.018	−9.373	−1.713	−0.546	−10.818
